# Dual-Attention-Based Block Matching for Dynamic Point Cloud Compression

**DOI:** 10.3390/jimaging11100332

**Published:** 2025-09-25

**Authors:** Longhua Sun, Yingrui Wang, Qing Zhu

**Affiliations:** 1 School of Information Science and Engineering, Qilu Normal University, No. 2 Wenbo Road, Jinan 250200, China; 2 Faculty of Information Technology, Beijing University of Technology, No. 100 Pingleyuan, Beijing 100124, China

**Keywords:** dynamic 3D point clouds, geometric compression, 3D reconstruction, motion estimation, motion compensation

## Abstract

The irregular and highly non-uniform spatial distribution inherent to dynamic three-dimensional (3D) point clouds (DPCs) severely hampers the extraction of reliable temporal context, rendering inter-frame compression a formidable challenge. Inspired by two-dimensional (2D) image and video compression methods, existing approaches attempt to model the temporal dependence of DPCs through a motion estimation/motion compensation (ME/MC) framework. However, these approaches represent only preliminary applications of this framework; point consistency between adjacent frames is insufficiently explored, and temporal correlation requires further investigation. To address this limitation, we propose a hierarchical ME/MC framework that adaptively selects the granularity of the estimated motion field, thereby ensuring a fine-grained inter-frame prediction process. To further enhance motion estimation accuracy, we introduce a dual-attention-based KNN block-matching (DA-KBM) network. This network employs a bidirectional attention mechanism to more precisely measure the correlation between points, using closely correlated points to predict inter-frame motion vectors and thereby improve inter-frame prediction accuracy. Experimental results show that the proposed DPC compression method achieves a significant improvement (gain of 70%) in the BD-Rate metric on the 8iFVBv2 dataset. compared with the standardized Video-based Point Cloud Compression (V-PCC) v13 method, and a 16% gain over the state-of-the-art deep learning-based inter-mode method.

## 1. Introduction

The impact of point cloud compression technology on real-world applications is profound, especially in fields such as autonomous driving [1], smart cities [2], and digitalization of cultural heritage [3]. Effective compression can significantly reduce storage and transmission costs, accelerate data flow, enable real-time processing and analysis, and thus promote industrial upgrading. Taking dense DPCs as an example, for a typical DPC dataset with a frame rate of 30 fps (frames per second), each 3D DPC consists of approximately 800,000 points, and transmitting the original point cloud video requires a bandwidth of approximately 1000 MB/s. Furthermore, for a mainstream LiDAR sensor with 64 threads, the generated DPCs per hour can reach a raw data volume of about 1 TB. Therefore, the massive amount of point cloud data brings great pressure to data processing, storage, and transmission, limiting large-scale, high-precision spatial information capture and hindering accurate scene perception.

In practical applications, the compression algorithm is usually used as a data pre-processing unit, first encoding data for transmission or storage, then decoding it for use. There is inevitable information loss during compression, which causes the loss of key geometric or semantic features, affecting the accuracy of subsequent tasks such as object recognition and scene understanding. The key to compression is balancing data reduction with reconstruction quality. Therefore, in addition to extracting data features as much as possible at the encoder, achieving high-quality point cloud reconstruction at the decoder is also an important aim. Consequently, some works adopt upsampling [4,5,6] or completion [7,8,9] approaches in the reconstruction module to maintain high visual fidelity and structural integrity even under lossy compression conditions.

According to different acquisitions, point clouds are usually divided into static and dynamic point clouds. DPCs usually consist of a sequence of static point clouds. In most practical applications, DPCs are more commonly used. Compared with static point clouds, DPCs not only have spatial redundancy but also temporal redundancy. Due to the irregular and highly non-uniform spatial distribution of points, removing spatiotemporal redundancy from DPCs remains extremely challenging. Therefore, we focus on the compression of DPC geometry by employing a specially designed end-to-end network.

Traditional DPC compression methods rely on projection algorithms [10,11]. For example, the Moving Picture Experts Group (MPEG) proposed Video-based Point Cloud Compression (V-PCC) [12], which projects DPCs into 2D geometry and texture videos and then uses the existing 2D image/video codec to compress the generated videos. Among these methods, V-PCC achieves the most satisfactory results. However, rule-based methods fail to fully capture the temporal dependencies due to the sparsity and non-uniform structure of DPCs. Other DPC compression methods rely on hand-crafted temporal context selection algorithms for inter-prediction. Some methods directly perform block-based matching in 3D space, utilizing existing partitioning and matching methods to find corresponding points between DPCs [13,14,15,16,17]. Recent research has begun to explore learning-based methods, which attempt to leverage the strong learning ability of networks to model the temporal correlation between DPC frames. These methods optimize feature extraction across entire datasets, achieving significant improvements over traditional rule-based algorithms. Usually, methods for point clouds are divided based on different representations: point-based methods [18,19,20], voxel-based methods [21,22,23], and octree-based methods [24,25,26,27]. However, directly extending these methods to DPCs still lacks applicability. Inspired by the ME/MC compression framework in 2D image/video compression methods, existing learning-based DPC compression methods attempt to model temporal dependence through an end-to-end ME/MC network [28]. The ME/MC compression framework first searches and calculates the motion vector of each point between the current frame and the reference frame, which is called “motion estimation”. The motion vector is then added to the points in the reference frame to achieve “motion compensation”, which predicts the points in the current frame. Subsequently, only the prediction residuals and a small number of motion vectors are transformed, quantized, and entropy-encoded, using temporal redundancy to significantly reduce the bit rate. As existing studies [29,30] based on the ME/MC framework still lack explicit ME/MC structures or rely on simplistic block-matching mechanisms, their motion estimation efficiency remains limited.

To address these limitations, we propose a learning-based DPC geometry compression framework that adaptively matches blocks between previously reconstructed and current frames. Our framework extracts hierarchical optical flow at different scales and produces more accurate inter-frame matching by analyzing the local similarity of geometry in latent spaces. The key contributions of this paper are as follows:

(1) We propose a multi-scale, end-to-end DPC compression framework, which jointly optimizes MC/ME, motion compression, and residual compression.
(2) The proposed hierarchical point cloud ME/MC scheme is a multi-scale inter-prediction scheme, which predicts motion vectors at different scales and improves inter-frame prediction accuracy.
(3) The designed dual-attention-based KNN block matching (DA-KBM) network efficiently measures feature correlation between points in adjacent frames. It strengthens the predicted motion flow and further improves the inter-prediction efficiency.

The paper is organized as follows: first, we summarize the most relevant works in Section 2. Then the proposed method is described in detail in Section 3. Furthermore, Section 4 analyzes the experimental results. Finally, the work is summarized in Section 5.

## 2. Related Works

This research is most closely related to the following research topics: rule-based point cloud compression and deep learning-based point cloud compression.

### 2.1. Rule-Based Point Cloud Compression

Traditional rule-based point cloud compression techniques are primarily categorized into 2D-projection-based approaches and 3D-based methods.

#### 2.1.1. 2D-Projection-Based Methods

Two-dimensional (2D) projection-based methodologies [10,11,12,31,32] capitalize on existing video codec technologies, with most research focusing on projection algorithm development and patch configuration optimization. For example, He et al. [10] implemented cubic projection techniques, while Zhu et al. [11] proposed a hybrid approach that combines view-based global projection with patch-based local projection. Furthermore, the Moving Picture Experts Group (MPEG) introduced a robust Video-based Point Cloud Compression (V-PCC) [12] standard specifically designed for Dynamic Point Cloud (DPC) compression. This standard synthesizes cubic projection, patch generation, and packing processes to produce geometry and texture maps. V-PCC demonstrates unparalleled performance, surpassing both 3D and alternative 2D projection-based compression techniques in terms of efficacy. However, the inherent distortion introduced by projection operations in 2D-based methods compromises inter-frame consistency and disrupts the spatial topology of the data, leaving significant room for improvement.

#### 2.1.2. 3D-Based Methods

3D-based methods can effectively maintain the geometric structure of point cloud space during compression. As the octree provides an efficient way to partition 3D space to represent point clouds, octree-based methods are the most widely used point cloud encoding approaches [33,34]. In these methods, the point cloud is first transformed into a volumetric representation and then recursively divided by the octree until it reaches the leaf nodes. The occupancy of the nodes is then compressed using an entropy context model. For DPC compression, 3D-based methods usually first explore inter-frame correspondence by block matching, followed by applying the ME/MC framework to multiple frames. Hong et al. [14] refined this paradigm by introducing a half-voxel refinement pattern, yielding optical-flow fields with sub-voxel accuracy between octree blocks. Subsequent studies have further advanced 3D motion estimation through alternative matching algorithms such as Iterative Closest Point (ICP) [16] and k-nearest neighbor (KNN) algorithms [15], each contributing complementary strategies to enhance the fidelity and robustness of inter-frame alignment under irregular sampling patterns. Thanou et al. [34] implemented an octree-based encoding method capable of predicting graph-encoded octree structures.

### 2.2. Learning-Based Point Cloud Compression

Usually, according to different representations of point clouds, learning-based point cloud compression methods are typically classified into four categories: (I) voxelization-based methods; (II) octree-based methods; (III) point-based methods; and (IV) sparse tensors-based methods.

#### 2.2.1. Voxelization-Based Methods

Voxelization-based methodologies dominated early research efforts in point cloud compression, as exemplified by the works [35,36]. These pioneering approaches typically transform point clouds into volumetric representations through voxelization, subsequently partitioning them into smaller cubic blocks of size 64 × 64 × 64 voxels. The compression process employs autoencoder architectures that utilize 3D convolutional operations to encode these blocks into compact latent representations. For model optimization, these methods predominantly implement specialized loss functions, particularly focal loss or weighted binary cross-entropy loss, to address the inherent data imbalance. However, a significant limitation of these approaches lies in their computational inefficiency, because most voxels are empty, resulting in wasted computation and memory overhead.

#### 2.2.2. Octree-Based Methods

Octree-based methodologies [24,25,26,27,37,38,39,40] have emerged as an efficient approach for point cloud representation, offering superior storage and computational efficiency. These techniques utilize sophisticated entropy context modeling to predict the occupancy probability of each node, conditioned on both its hierarchical dependencies (parent nodes) and spatial relationships (neighboring nodes). The evolution of these methods demonstrates progressive advancements in context modeling: OctSqueeze [24] and MuSCLE [25] pioneered the use of Multi-Layer Perceptrons (MLPs) to capture and exploit the hierarchical dependencies between parent and child nodes. Building upon this foundation, VoxelContextNet [26] expanded the context modeling paradigm by incorporating not only parent and neighbor information but also integrating voxelized neighborhood points for enhanced probability estimation. The most recent advancement, OctAttention [27], represents a significant leap forward by implementing a large-scale transformer-based context attention module, which substantially increases the receptive field for occupancy code probability estimation. These lossless encoding methods have demonstrated particularly impressive performance on sparse LiDAR-based point clouds, establishing new benchmarks in the field. However, octree-based methods remain difficult to apply to dense point cloud compression.

#### 2.2.3. Point-Based Methods

Point-based methodologies represent a distinct paradigm in point cloud processing, maintaining the original point cloud representation without resorting to voxelization or other structural transformations. These approaches predominantly leverage PointNet [41] and its enhanced variant PointNet++ [42] architectures, which directly process raw point clouds through point-wise fully connected layers. The typical implementation involves patch-based processing, where farthest point sampling is employed for efficient subsampling, combined with k-nearest neighbor (KNN) search algorithms to establish per-point feature embeddings, ultimately constructing an MLP-based autoencoder framework. However, as demonstrated in several studies [43,44,45], these point-wise models exhibit notable limitations, particularly in coding efficiency, which remains suboptimal compared to alternative approaches. Moreover, these methods struggle with scalability issues, showing limited generalization capability when applied to large-scale dense point clouds. An additional drawback lies in the computational overhead, since they require extensive pre- and post-processing, which significantly reduces overall encoding efficiency.

#### 2.2.4. Sparse Tensors-Based Methods

Recent sparse convolution-based methods [28,29] first convert the raw point-cloud sequences into Minkowski sparse tensors [46], which process dense point clouds by leveraging sparsity for efficient, deep network processing of large-scale data, enhancing local and global 3D feature extraction. However, although effective for static point clouds, they face challenges in dynamic compression tasks. Akhtar et al. [28] introduced multi-scale feature fusion without explicit motion estimation/compensation (ME/MC), later addressed by Fan et al. [29] through D-DPCC’s feature-domain ME/MC and 3D adaptive interpolation. However, D-DPCC’s single ME/MC pass leaves temporal dependencies underutilized. Our work advances this by developing a sparse convolutional autoencoder with inter-frame prediction, analogous to P-frames in video encoding, using decoded frames for subsequent frame encoding.

## 3. Proposed Method

### 3.1. Overview of Proposed DPCs Compression Network

**Figure 1:**
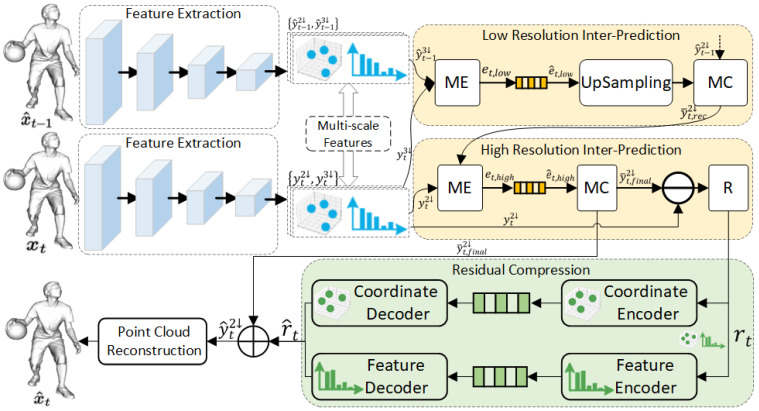
The proposed inter-frame encoding method with multi-scale motion estimation and motion compensation.

Figure 1 illustrates the overall architecture of the proposed dynamic compression approach. The network consists of five collaboratively designed modules: feature extraction module, low-resolution inter-prediction module, high-resolution inter-prediction module, residual compression module, and point-cloud reconstruction module. Based on the recent work published by Wang et al. [22], the raw point-cloud sequences are first converted into Minkowski sparse tensors [46]. To elaborate, we define two sequential point cloud frames as $x_{t-1}=\left\{C\left(x_{t-1}\right)\;F\left(x_{t-1}\right)\right\}$ and $x_{t}=\left\{C\left(x_{t}\right)\;F\left(x_{t}\right)\right\}$, where $C(x_{t-1})$ and $C(x_{t})$ correspond to the coordinates of occupied points, $F(x_{t-1})$ and $F(x_{t})$ represent the associated features with values of one corresponding to occupied points.

The designed network accepts the encoding frame $x_{t}$ together with the prior decoded point cloud ${\hat{x}}_{t-1}$ (serving as a reference) as inputs. These are subsequently encoded by the feature extraction module into latent representations $y_{t}$ and ${\hat{y}}_{t-1}$ at multiple scales. Specifically, $y_{t}^{2↓}$/${\hat{y}}_{t-1}^{2↓}$ and $y_{t}^{3↓}$/${\hat{y}}_{t-1}^{3↓}$ represent the latent features downsampled by factors of 2 and 3, respectively. The low-resolution inter-prediction module employs the $y_{t}^{3↓}$ and $\hat{y}t^{3↓}$ as its inputs. This module produces two primary outputs: the low-resolution flow embedding $\hat{e}t,l$ and an initial reconstruction ${\bar{y}}^{2↓}t,rec$ for the frame $yt^{2↓}$. The latter output, ${\bar{y}}^{2↓}t,rec$, acts as the reference for the subsequent high-resolution inter-prediction module, which is responsible for generating the high-resolution flow embedding $\hat{e}t,h$ and the final prediction ${\bar{y}}_{t,final}^{2↓}$. The residual compression module handles the compression and decompression of the residual $r_{t}$ between $y_{t}^{2↓}$ and ${\bar{y}}_{t,final}^{2↓}$. During decoding, the decompressed residual $r_{t}$ is summed with ${\bar{y}}^{2↓}t,final$, yielding the reconstructed latent representation ${\hat{y}}^{2↓}t$. This representation is subsequently processed by the reconstruction module, which employs an upsampling network to produce the current frame $x_{t}^{\prime }$. The specific architectures of all modules will be detailed in the sections that follow.

### 3.2. Feature Extraction Module

The architecture of the feature extraction module, adapted from [30], is shown in Figure 1. It consists of sequentially connected down-sampling blocks that progressively reduce spatial redundancies, thereby producing the latent features $y_{t}$ and $\hat{y}t-1$ for the frames $xt^{\prime }$ and ${\hat{x}}_{t-1}$.

**Figure 2:**
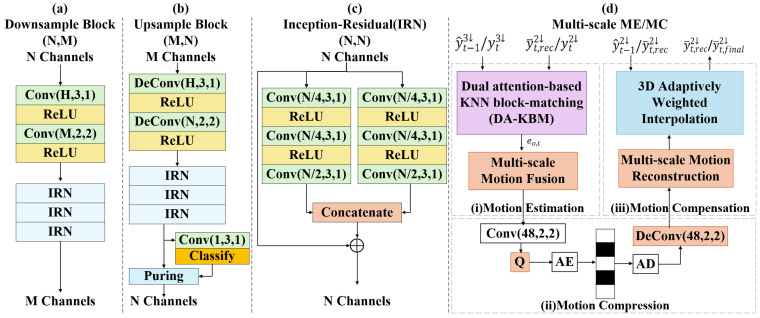
(**a**–**c**) Structure of the feature extraction modules. (**d**) Structure of the multi-scale ME/MC.

This paper’s feature extraction module contains two downsampling blocks to produce the 2× and 3× down-sampled latent feature $y_{t}^{2↓}$/$y_{t-1}^{2↓}$ and $y_{t}^{3↓}$/$y_{t-1}^{3↓}$. Details of the downsampling block are shown in Figure 2a.

### 3.3. Multi-Resolution-Based Inter-Prediction

A designed multi-scale ME/MC scheme is employed within the inter-prediction module, as illustrated in Figure 1. Furthermore, the detailed structure of the multi-sale ME/MC is shown in Figure 2d. To further improve point motion estimation accuracy, we proposed a dual attention-based KNN block-matching (DA-KBM) network in the motion estimation module. It measures the temporal interrelationship between the latent features $y_{t}$ and ${\hat{y}}_{t-1}$ via a designed dual-attention scheme to generate the original flow embedding $e_{o,t}$. Subsequently, the latent embedding $e_{o,t}$ is forwarded to the Multi-scale Motion Fusion (MMF) module [29] to enhance the generated motion embedding ${\hat{e}}_{t}$. A motion compression module is then followed, which utilizes an auto-encoder network and leverages a non-parametric, fully factorized density model [47] to jointly compress and reconstruct the motion embedding ${\hat{e}}_{t}$. To perform motion compensation [29], the decompressed flow embedding ${\hat{e}}_{t}$ is forwarded to the Multi-scale Motion Reconstruction (MMR) module. It hierarchically recovers fine-grained optical-flow components from the coarse one, yielding the final 3D point prediction.

#### 3.3.1. Dual Attention-Based KNN Block Matching (DA-KBM)

**Figure 3:**
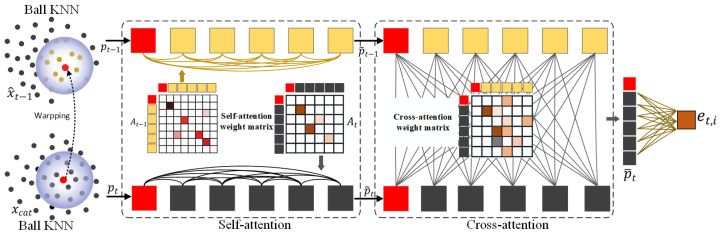
Network of motion estimation via dual attention-based KNN block matching (DA-KBM).

The motion-estimation scheme originally proposed in D-DPCC [29] is constrained by its shallow, two-layer SparseCNN architecture, which is insufficient for establishing accurate block-level correspondences across frames. To overcome this limitation, we propose a dual-attention KNN-based block-matching (DA-KBM) network that substantially refines motion estimation accuracy. As illustrated in Figure 3, DA-KBM first employs ball-KNN to collect local spatio-temporal neighbors within a spherical region; two cascaded inter-frame attention modules then weigh these neighbors according to both geometric proximity and feature affinity, generating soft correspondences rather than hard assignments. This design enables the network to capture subtle, non-rigid motions that the original two-layer SparseCNN in D-DPCC [29] fails to resolve, yielding significantly more precise inter-frame block correspondences. It is important to note that the proposed network integrates the referenced point cloud with the current encoding point cloud to create the initial representation. This integration facilitates the concurrent aggregation of information from blocks across both frames. Then, for the sparse tensors, the operator of concatenation can be defined as:

(1) $$y_{cat,u}=\left\{\begin{array}{cc} y_{t,u}⊕y_{t-1,u} & u\in C\left(y_{1}\right)\cap C\left(y_{t-1}\right)\\ y_{t,u}⊕0 & u\in C\left(y_{t}\right),u\notin C\left(y_{t-1}\right)\\ 0⊕y_{t-1,u} & u\notin C\left(y_{t}\right),u\in C\left(y_{t-1}\right) \end{array}\right.$$

where $y_{t}$ and $y_{t-1}$ are latent features of the reference and current frames, $C(y_{t})$ and $C(y_{t-1})$ are the coordinate tensors. ⊕ is the operator of concatenation for features.

Formally, for *i*-th point in $y_{t}$ (denoted as $p_{t,i}$), the network first performs a spherical K-nearest-neighbor (ball-KNN) search to collect two local neighborhoods: $N\in y_{t-1}$ and $N_{cat}\in y_{cat}$, both restricted to a radius r and capped at K = 9 points in our experiments. These neighbors are then processed by a dual-attention mechanism: Self-attention operates within each neighborhood independently. An attention weight matrix $A^{self}\in R^{K\times K}$ is computed from the attribute vectors via:

(2) $$\alpha_{uv}^{self}=softmax_{u}\left(\frac{{\left(W_{Q}a_{u}\right)}^{T}\left(W_{K}a_{v}\right)}{\sqrt{d_{k}}}\right)$$

followed by a weighted aggregation $h_{u}=\sum_{v}\alpha_{uv}^{self}\left(W_{V}a_{v}\right)$. Cross-attention aligns the updated representations of $N_{cat}$ and $N_{t-1}$. A second weight matrix $A^{corss}\in R^{K\times K}$ is computed between the two sets, enabling inter-frame feature fusion: $g_{i}=\sum_{q\in N_{t-1}}\alpha_{iq}^{corss}\left(W_{V}^{\prime }h_{q}\right)$. The resulting fused descriptor $g_{i}$ is finally projected through a shared MLP to yield the per-point flow embedding $e_{t,i}=MLP_{\theta }\left(g_{i}\right)$, which compactly encodes the correspondences required for accurate motion estimation.

#### 3.3.2. Motion Compensation Module

The estimated motion flow $e_{t}$ with the shape of $R^{N\times 64\times 3}$ is composed of 64 individual motion flows, which corresponds to each channel of the latent feature $y_{t}^{2↓}\in R^{N\times 64}$. As a result, every channel can independently find its match in the referenced point cloud, which contributes to a richer temporal context. The process starts with the network warping the coordinates in $y_{t}^{2↓}$ separately for each channel:

(3) $$u_{w}^{\left(i\right)}=u+e_{t,u}^{\left(i\right)},\;\forall u\in C\left(y_{t}^{2↓}\right)$$

where $C(y_{t}^{2↓})$ represents the coordinates of $y_{t}^{2↓}$, u is an arbitrary coordinate within $C(y_{t}^{2↓})$, i denotes the channel index. The term $u_{w}^{\left(i\right)}$ corresponds to the warped coordinate of u within the *i*-th channel, and $m_{t,u}^{\left(i\right)}=\left(▵x,▵y,▵z\right)$ is formulated as the *i*-th channel motion flow vector at position u. To accommodate the sparse distribution of point cloud data and ensure differentiability in the prediction process, the 3D Adaptively Weighted Interpolation (3DAWI) framework [29] is employed to execute motion compensation, defined by:

(4) $${\bar{y}}_{t,u}^{\left(i\right)}=\frac{\sum_{v\in V(u_{w}^{i})}d{\left(u_{w}^{i},v\right)}^{-1}\cdot y_{ref,v}^{\left(i\right)}}{max(\sum_{v\in v(u_{w}^{\left(i\right)})}d{\left(u_{w}^{i},v\right)}^{-1},\alpha )},\forall u\in C\left(y_{t}^{2↓}\right)$$

where $\theta (u^{\left(i\right)}w)$ signifies the set of three nearest spatial neighbors around the warped point $u^{\left(i\right)}w$, $d{\left(u_{w}^{i},v\right)}^{-1}$ corresponds to the inverse of the Euclidean distance. It measures the relationship between $u^{\left(i\right)}w$ and a neighboring point *v* by the Euclidean distance. $y{ref,v}^{\left(i\right)}$ indicates the *i*-th channel feature values from the referenced point cloud at the corresponding position *v*. The reference frame $y_{ref}$ is defined as $y_{t-1}^{2↓}$ in the low-resolution case and as $\bar{y}t,ini$ in the high-resolution inter-prediction stage. Additionally, a penalty coefficient α is incorporated to adaptively reduce the influence of isolated points. Crucially, $C(yt^{2↓})$, as referenced in Equations (1) and (2), undergoes lossless encoding.

### 3.4. Residual Compression Module

The auto-encoder (AE) architecture adopted by the residual compression module (Figure 2) encodes the geometry-feature residual $r_{t}=y_{t}^{2↓}-{\bar{y}}_{t,final}^{2↓}$ into a compact latent representation. On the encoder side, a parametric analysis transform first applies a sparse down-sampling block [22] followed by a 3D sparse convolution, yielding the latent representation $l_{rt}=\left\{C\left(y_{t}^{3↓}\right),F\left(l_{r_{t}}\right)\right\}$.

As shown in Figure 2, an auto-encoder (AE) network is employed in the compression module of residual features. It encodes the residual $r_{t}$ between $y_{t}^{2↓}$ and the final prediction ${\bar{y}}_{t,final}$. On the encoder side, the parametric analysis transform, comprising a downsample block [22] and a convolution layer, converts $r_{r}$ into a more compact latent representation $l_{rt}=\left\{C\left(y_{t}^{3↓}\right),F\left(l_{r_{t}}\right)\right\}$. The coordinate $C(y_{t}^{3↓})$ is lossless compressed via the G-PCC v14 algorithm [12], while the feature tensor $F(l_{r_{t}})$ undergoes quantization followed by entropy coding based on a fully-factorized density model [47]. During decoding, a symmetric parametric synthesis transform—implemented by a sparse up-sampling block—reconstructs the residual $r_{t}$. The fused flow embedding $e_{t}$ undergoes an analogous compression pipeline: a single sparse convolution layer performs analysis encoding, and its transpose counterpart performs synthesis decoding, sharing the same quantization and entropy-modeling strategy as the residual branch.

### 3.5. Point Cloud Reconstruction Module

There are cascaded upsampling modules composing the reconstruction module. As shown in Figure 2b, the reconstruction module is symmetric to the feature extraction module. These blocks hierarchically reconstruct the current frame ${\hat{x}}_{t}$ from the reconstructed latent feature $y_{t}^{\prime }$. Furthermore, to estimate the occupancy probability of each point, an additional sparse convolution layer with a single output channel is incorporated. Based on these probabilities, a subsequent pruning operation [22] is then performed to eliminate outliers.

### 3.6. Loss Function

As shown below, the rate-distortion loss function serves as the objective for optimizing the proposed end-to-end network:

(5) L=R+λD

where R is the rate cost, which is the bits per point (bpp) in the coding. In this work, the rate R is composed of three parts: the bpp of coding low-resolution flow embedding $R_{l}$ the high-resolution flow embedding $R_{h}$, and the cost of compressing the feather residual $R_{resdual}$. D means distortion, which measures the reconstruction error between the ground-truth point cloud $x_{t}$ and the reconstructed point cloud ${\hat{x}}_{t}$. The parameter λ adjusts the balance between bit-rate and distortion. Each part of R is computed via:

(6) $$R_{\tilde{F}}=\frac{1}{N}\sum_{i}-log_{2}\left(P_{\tilde{F}|\psi }\right)$$

where the $\tilde{F}$ is the quantized latent feature. The variable N corresponds to the point count in the original frame, and the parameter i identifies the index of channels within the latent feature. During the training state, adding uniform noise w∼U(−0.5,0.5) will serve as a differentiable approximation of the quantization operation. The utilized fully factorized entropy model [47] can then estimate the probability distribution of the encoding feature $\tilde{F}$, which is denoted as: $P_{\tilde{F}|\psi }$. There, the parameter ψ is a learnable parameter. Finally, the Binary Cross Entropy (BCE) is employed to measure the distortion of the reconstructed point cloud:

(7) $$D_{BCE}=\frac{1}{N}\sum_{v}-\left(O_{v}logp_{v}+\left(1-O_{v}\right)log\left(1-p_{v}\right)\right)$$

where $O_{v}$ denotes the ground truth occupancy indicator, specifying whether the point vs. is truly occupied in the original point cloud. During multi-scale reconstruction, the binary cross-entropy (BCE) losses from all upsampling blocks are averaged to compute the final distortion:

(8) $$D=\frac{1}{K}\sum_{k=1}^{K}D_{BCE}^{k}$$

where k is the scale index.

## 4. Experiments

### 4.1. Datasets

Training Dataset.The proposed model is trained on the Owlii dynamic character point-cloud dataset [48], which comprises four sequences each containing 600 frames, captured at 30 fps (total duration 20 s per sequence). To mitigate training-time memory consumption and to demonstrate scalability, the original 11 bits precision point coordinates are uniformly quantized to 10 bits.

Test Dataset. Comprehensive evaluation is conducted on the 8i Voxelized Full Bodies v2 (8iVFBv2) [49] dataset released by the MPEG Point-Cloud Standardization Group. This benchmark contains four sequences of 300 frames, acquired at 30 fps (10 s each).

### 4.2. Training Details

To span the target rate–distortion region, six independent models are trained by assigning the Lagrange multiplier λ∈{3,4,5,6,7,10}, yielding operating points approximately centered at {0.075,0.10,0.15,0.20,0.25,0.30} bits per point (bpp). The model was optimized using the Adam algorithm, whose learning rate is decayed by a factor of 0.7 every 15 epochs. Each model is trained for a total of 50 epochs in two phases: during the first five epochs, λ is fixed at 20 to accelerate convergence of the reconstruction pathway; thereafter, λ is switched to its designated value for rate-distortion optimization. A batch size of one is employed, and the experimental setup utilized a solitary NVIDIA GeForce RTX 3090 GPU(made by NVIDIA, Santa Clara, CA, USA), which provided 24 GB of memory for all trials.

### 4.3. Baseline Settings

To evaluate the compression performance, the proposal was compared with several SOTA point cloud compression methods, such as: MPEG standard test model v13: MPEG G-PCC (octree & trisoup) [50] and MPEG V-PCC [12], as well as deep learning-based methods PCGCv1 [23], PCGCv2 [22] and D-DPCC [29]. For a fair comparison, we re-train the learning-based methods. For objective evaluation, the cost of bits in compression is measured by bpp, and reconstruction fidelity is quantified by the metrics of D1 PSNR (point-to-point distance) and D2 PSNR (point-to-plane). The peak signal value of these two metrics is set as 1023 for the 10-bit quantized 8iVFBv2 [49] data. Rate–distortion (R-D) curves are plotted and Bjøntegaard Delta-rate (BD-rate) gains are computed with respect to prior state-of-the-art codecs, expressing the average percentage reduction in bit rate at equivalent objective quality. In line with common practice, we use PCGCv2 [22] to intra-code the first frame of each sequence, after which all subsequent frames are predictively coded (P-frames) relative to the immediately preceding reconstructed frame.

### 4.4. Experimental Results

**Figure 4:**
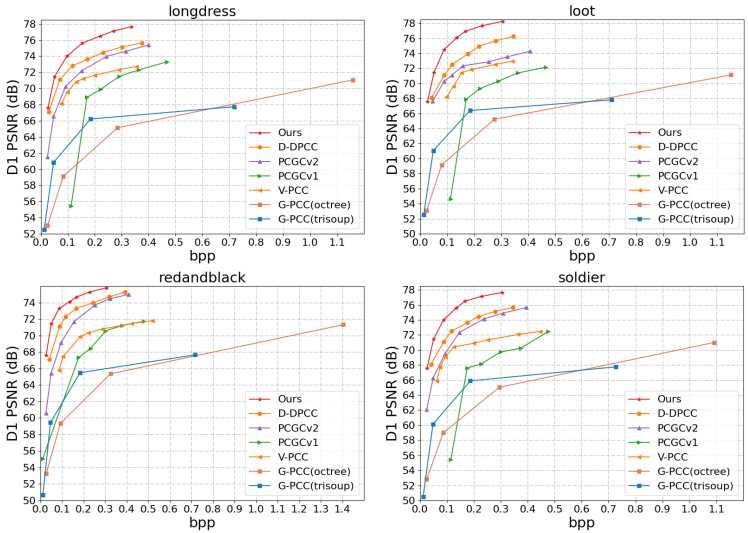
Comparison of R-D curves based on D1 PSNR.

**Figure 5:**
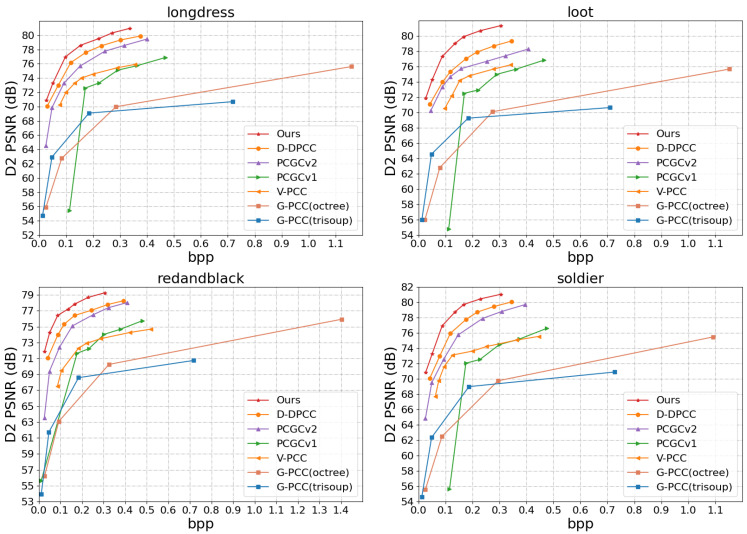
Comparison of R-D curves based on D2 PSNR.

**Figure 6:**
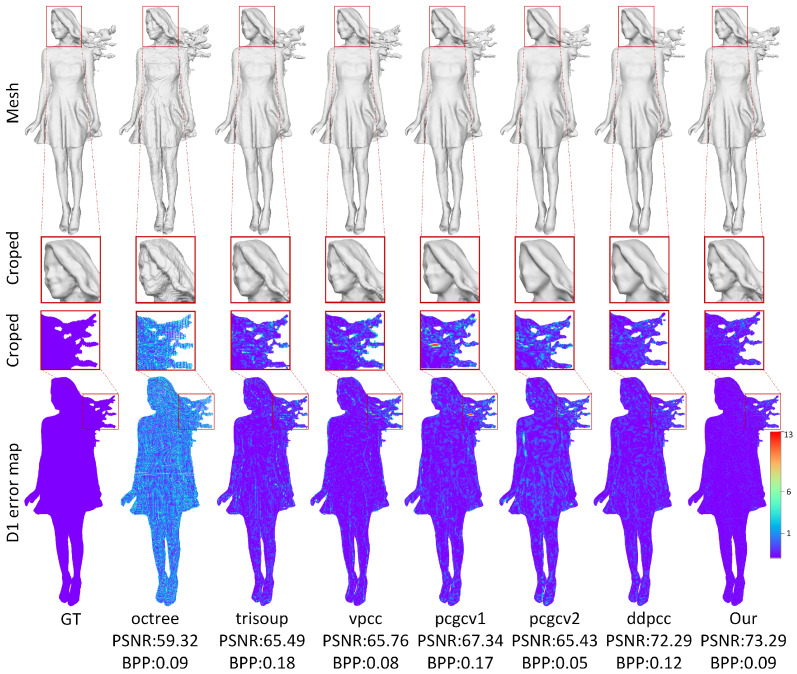
Visual comparison of reconstructed sequence “Redandblack”.

**Figure 7:**
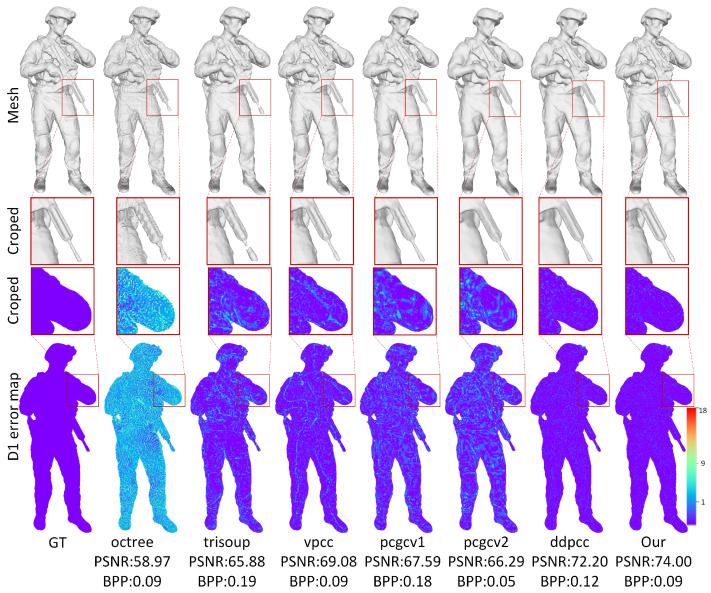
Visual comparison of reconstructed sequence “Soldier”.

This work evaluates the proposed method from both quantitative and qualitative perspectives. The quantitative results are presented in Figure 4 and Figure 5, as well as Table 1 and Table 2, while the qualitative results are illustrated in Figure 6 and Figure 7.

Objective Evaluation. Using the D1-PSNR and D2-PSNR quality metrics, the rate-distortion performance on the 8iVFBv2 [49] test sequences is illustrated in Figure 4 and Figure 5, respectively. The associated comparisons about BD-rate and BD-PSNR are also displayed in Table 1 and Table 2. From Figure 4 and Figure 5 we can see that the most deep learning-based methods benefit from the powerful learning ability of the network and are generally superior to standardized methods [12]. Furthermore, V-PCC [12] performs better on dense point clouds compared to the G-PCC method. It also outperforms the block-based end-to-end encoding method PCGCv1 significantly in D1 PSNR, and achieves comparable results in D2PSNR metrics. PCGCv2 [22] is currently a highly competitive intra-frame encoding method, and in the vast majority of cases, it outperforms other compared intra-frame encoding methods (G-PCC [50], V-PCC [12], PCGCv1 [23]). D-DPCC [29] not only removes spatial redundancy through down-sampling, but also utilizes inter-frame correlation for temporal prediction. compared to other intra-frame encoding methods, D-DPCC [29] effectively remove redundant information and achieving more efficient encoding performance. The method proposed in this article also utilizes both spatial and temporal dimensions to remove redundant information, and further optimizes inter-frame prediction. Compared with D-DPCC [29], it achieves significant improvement on the R-D curve.

As shown in Table 1 and Table 2, the proposed DPCs compression method achieves 70.92% (point-to-point distance) and 62.63% (point-to-plane distance) average BD-rate gains and 7.11 dB (point-to-point distance) and 5.77 dB (point-to-plane distance) average BD-PSNR gains on four sequences, respectively. Compared with the SOTA learning-based method D-DPCC [29], the proposal obtains 28.24% (point-to-point distance) and 16.37% (point-to-plane distance) average BD-rate gains, which is due to the proposed multi-resolution-based inter-prediction module and the DA-KBM module. This also indicates that the two proposed modules further explore the effective temporal context and improve the accuracy of inter-prediction.

Visual Comparisons. Qualitative evaluations are presented in Figure 6 and Figure 7, where the “redandblack” and “solid” sequences are reconstructed at comparable approximate bit rates. The spherical Poisson surface reconstruction method [51] was adopted to perform surface fitting on the reconstructed point cloud, as shown in the first row of Figure 6 and Figure 7. Furthermore, the cropped areas are enlarged in the second and third rows to enable a more comprehensive comparison of the reconstruction quality of different methods. From the results of mesh reconstruction, it can be seen that at approximate bit rates, G-PCC [50] suffers from pronounced structural erosion, whereas PCGCv1 [23] and PCGCv2 [22] produce overly smoothed surfaces that sacrifice fine-grained detail. Inter-frame predictive D-DPCC [29] and the proposed method consistently deliver the highest geometric fidelity, with the latter preserving markedly richer structure.

### 4.5. Ablation Study

**Figure 8:**
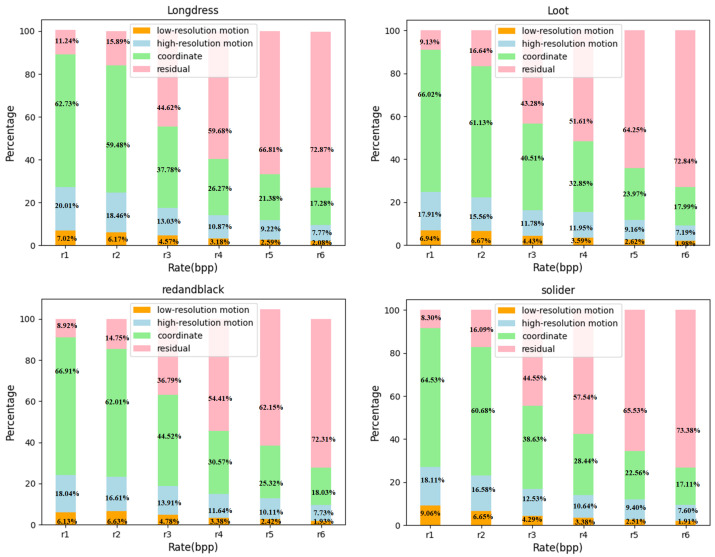
Composition of bit rate with different λ.

Ablation study on Bit-rate components. Figure 8 presents the bit-rate apportionment among the individual codec components across the six trained λ settings. It is evident that the motion-related bitstreams consume only a minor fraction of the overall rate. Specifically, the low-resolution motion stream accounts for merely 2–7% of the total bitrate, whereas the high-resolution motion stream demands 7–20%. As λ increases, the relative bitrate allocated to motion information exhibits a monotonic decline, while the share devoted to residual coding grows commensurately. A larger λ penalizes distortion more heavily, thereby driving the model to preserve richer geometric and textural details, indicating that the residual features contain more detailed information, which is necessary for reconstructing high-quality point clouds. Within the residual stream, the coordinate component’s bitrate fraction decreases with rising λ, whereas the feature component’s fraction increases. This inverse trend corroborates the intuition that, at higher fidelity settings, investing additional bits in expressive feature channels yields greater perceptual and numerical gains than expending them on further refinement of already coarse coordinates.

**Figure 9:**
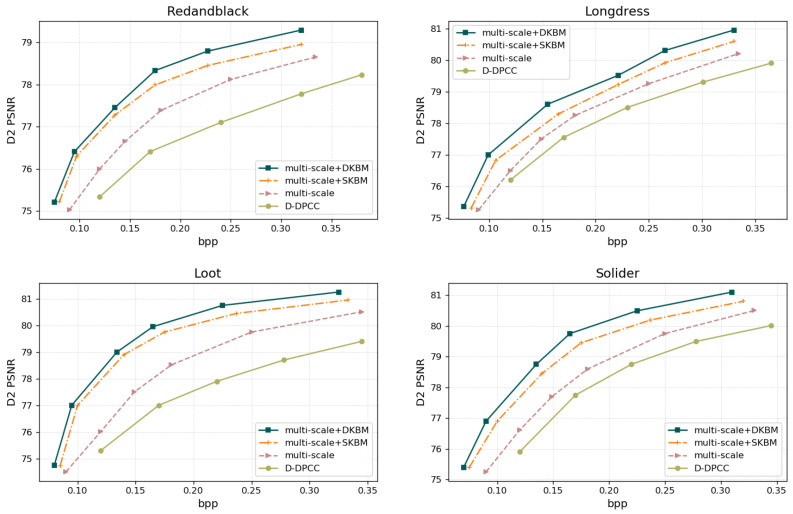
Performance of ablation study on different components. Comparison of R-D curves based on D1 PSNR.

Ablation study on different components. Figure 9 reports the rate–distortion curves obtained from the ablation study on the different components, wherein each curve corresponds to a variant of the proposed architecture with the module either retained or systematically removed. Using D-DPCC [29] as the baseline, we incrementally equip it with (i) the proposed multi-resolution inter-frame prediction module (denoted “multi-scale”) and (ii) the dual-attention block matching module (denoted “DKBM”). To further prove the effectiveness of DKBM, an experiment with a single-attention block matching module (denoted “SKBM”) is conducted. Both the “multi-scale” and DKBM modules substantially refine motion estimation, yielding commensurate gains in coding efficiency. As evidenced in Figure 9, the “multi-scale” module already delivers a noticeable R-D improvement relative to the D-DPCC [29] baseline by sequentially predicting the current frame’s latent representation across multiple scales. Augmenting this multi-scale structure with the SDKM module further elevates performance: the attention mechanism fuses spatio-temporal features within local matching neighborhoods across the two frames, producing a more accurate motion-flow field. The DKBM module further improves the model’s rate distortion performance, indicating that this dual attention module can more fully learn inter-frame correlations and improve the accuracy of motion flow prediction.

**Figure 10:**
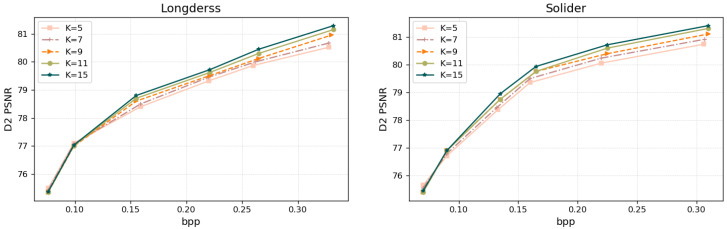
Performance analysis of the number of neighbors K. Comparison of R-D curves based on D1 PSNR.

Ablation study on different K. Note that the number of neighbors K is also an important parameter of the proposed DKBM module. The ablation results are shown in Table 3 and Figure 9. In the experiments, the K is set as K ={5,7,9,11,13,15} to have more neighbors for information aggregation, with the performance improvement displayed in Table 3 demonstrating its efficiency compared with K = 5 and K = 7. Figure 10 shows that the increase in K value has little effect at the low bit rate. At the high bitrate, more effective temporally correlated points are explored, and the increase in K value further improves the accuracy of motion estimation. However, the increase in K value to some extent improves the encoding and decoding time.

Ablation study on the number of attention heads. Table 4 shows the BD-PSNR results with different numbers of attention heads of the DKBM module. As the number of attention heads increases, the encoding efficiency improves, indicating that multi-head attention can improve inter-block matching efficiency by exploring deeper latent features. However, as shown in the experimental results, the improvement in encoding performance is not as significant when the number of heads is 3 as when it is 2. It can be predicted that as the number of heads increases, the impact on encoding performance will also tend to saturate.

### 4.6. Model Complexity

Finally, we conduct a computational-complexity analysis. All results reported in Table 5 were measured on an Intel Xeon Gold 6226R CPU (maded by Intel, Santa Clara, CA, USA) (2.90 GHz) and an NVIDIA GeForce RTX 3090 GPU (24 GB). V-PCC [12] incurs the highest latency because its patch segmentation, packing, and depth-image generation stages are inherently sequential and exhibit limited parallelism. G-PCC [12] achieves the lowest runtime, but this efficiency is accompanied by the poorest reconstruction quality, as corroborated by the quantitative and qualitative results presented earlier. For the learning-based methods, PCGCv1 [23] achieves the most time, due to the design of a hyper-prior model, which also leads to the highest FLOPs and most params. PCGCv2 [22] is the first work using sparse convolution to extract 3D point cloud features, which achieves the fastest speed of encoding and decoding times. Compared with the recent work D-DPCC [29], the proposed method introduces an additional 35% overhead (2.57 s vs. 1.67 s per frame), attributable to the multi-resolution inter-frame predictor and the dual-attention motion-estimation module. Critically, the modest complexity increase is offset by an average 28.24% BD-rate reduction and a 2.54 dB D2-PSNR improvement, rendering the overall complexity–performance trade-off acceptable.

## 5. Conclusions

The proposed method is a multi-scale-based, end-to-end DPC compression framework, jointly optimizing MC/ME, motion compression, and residual compression. The first key contribution is the design of a hierarchical ME/MC scheme, which predicts motion vectors at different scales to improve inter-frame prediction accuracy. The second contribution is the design of a dual-attention-based KNN block matching (DA-KBM) network, which efficiently measures the feature correlation between points in adjacent frames. It strengthens the predicted motion flow and further enhances inter-prediction efficiency. SOTA evaluation confirmed that the proposed method achieves superior reconstruction accuracy at a lower bitrate cost compared with other methods. The ablation study validated the importance of the hierarchical ME/MC scheme and the dual-attention-based KNN block matching (DA-KBM) network, as well as their positive impact on encoding efficiency. Although this work has achieved certain algorithmic improvements in dynamic point cloud encoding, limitations remain. For example, on the decoding side, point cloud reconstruction is currently achieved through a simple cascaded upsampling module, which does not fully utilize the powerful learning capability of deep learning networks. Future work will focus on refining and redesigning the reconstruction network to further improve reconstruction quality. Future iterations may also explore the temporal correlation of large-scale scene sequence point clouds obtained by LiDAR within the ME/MC framework, while incorporating semantic feature encoding to enable efficient encoding and decoding for specified visual tasks.

## Figures and Tables

**Table 1:** BD-Rate results against the SOTA methods using D1 PSNR and D2 PSNR.

Dataset	Sequence	BD-Rate with D1 PSNR (dB)					
		G-PCC (octree)	G-PCC (trisoup)	V-PCC	PCGCv1	PCGCv2	D-DPCC
8iVFBv2	soldier	−98.21%	−96.28%	−76.87%	−93.31%	−43.09%	−33.45%
	longdress	−97.32%	−94.24%	−73.43%	−83.76%	−31.25%	−26.57%
	loot	−98.58%	−95.57%	−78.87%	−93.12%	−43.55%	−32.89%
	redandblack	−96.70%	−93.37%	−75.93%	−84.21%	−41.58%	−20.03%
Average with D1		−97.70%	−94.86%	−76.27%	−88.60%	−39.86%	−28.24%
Dataset	Sequence	BD-Rate with D2 PSNR (dB)					
		G-PCC (octree)	G-PCC (trisoup)	V-PCC	PCGCv1	PCGCv2	D-DPCC
8iVFBv2	soldier	−93.40%	−92.03%	−71.23%	−70.35%	−33.55%	−16.87%
	longdress	−92.68%	−91.52%	−68.58%	−68.73%	−27.83%	−12.33%
	loot	−93.78%	−92.42%	−73.34%	74.03%	−45.37%	−23.89%
	redandblack	−91.98%	−90.28%	−72.77%	−71.28%	−22.45%	−12.38%
Average with D2		−92.96%	−91.56%	−71.48%	−71.09%	−32.34%	−16.37%

**Table 2:** BD-PSNR results against the SOTA methods using D1 PSNR and D2 PSNR.

Dataset	Sequence	BD-PSNR with D1 PSNR (dB)					
		G-PCC (octree)	G-PCC (trisoup)	V-PCC	PCGCv1	PCGCv2	D-DPCC
8iVFBv2	soldier	13.17	10.37	5.37	9.31	3.63	2.92
	longdress	12.48	9.76	4.63	8.35	2.73	2.44
	loot	15.02	10.07	5.54	9.28	3.68	2.78
	redandblack	10.73	9.33	5.05	8.42	3.55	2.03
Average with D1		12.85	9.88	5.15	8.84	3.39	2.54
Dataset	Sequence	BD-PSNR with D2 PSNR (dB)					
		G-PCC (octree)	G-PCC (trisoup)	V-PCC	PCGCv1	PCGCv2	D-DPCC
8iVFBv2	soldier	11.65	10.11	4.13	4.07	3.03	1.63
	longdress	10.92	9.38	4.27	4.43	2.55	1.33
	loot	13.87	10.18	4.56	4.98	3.88	2.42
	redandblack	9.75	9.36	4.32	4.16	2.13	1.38
Average with D2		11.54	9.75	4.32	4.41	2.89	1.69

**Table 3:** Ablation study results on parameter K (the number of neighbors). BD-Rate results against the baselines D-DPCC, using D1 PSNR and D2 PSNR.

Dataset	Sequence	BD-Rate with D1 PSNR (dB)					
		K = 5	K = 7	K = 9	K = 11	K = 13	K = 15
8iVFBv2	soldier	−30.22%	−31.45%	−33.45%	−34.72%	−35.57%	−36.34%
	longdress	−23.16%	−24.38%	−26.57%	−28.31%	−29.27%	−29.96%
	loot	−29.88%	−30.62%	−32.89%	−33.97%	−35.43%	−36.21%
	redandblack	−17.08%	−18.11%	−20.03%	−21.47%	−22.53%	−23.03%
Average with D1		−25.08%	−26.14%	−28.24%	−29.61%	−30.70%	−31.39%
Dataset	Sequence	BD-Rate with D2 PSNR (dB)					
		K = 5	K = 7	K = 9	K = 11	K = 13	K = 15
8iVFBv2	soldier	−13.07%	−14.23%	−16.87%	−17.43%	−18.26%	−19.17%
	longdress	−9.03%	−10.27%	−12.33%	−13.66%	−14.57%	−15.81%
	loot	−20.02%	−21.83%	−23.89%	−24.21%	−25.37%	−26.09%
	redandblack	−9.43%	−10.71%	−12.38%	−13.17%	−14.65%	−15.20%
Average with D2		−12.89%	−14.26%	−16.37%	−17.12%	−18.12%	−18.89%
Average Coding Time		2.21	2.36	2.57	3.10	3.90	4.60

**Table 4:** Ablation study results on the number of attention heads of the DKBM module. BD-PSNR results against the baselines D-DPCC, using D1 PSNR.

Dataset	Sequence	BD-Rate with D1 PSNR (dB)		
		Heads = 1	Heads = 2	Heads = 3
8iVFBv2	soldier	2.92	3.21	3.27
	longdress	2.44	2.73	2.79
	loot	2.78	2.92	3.01
	redandblack	2.03	2.25	2.32
Average with D1		2.54	2.77	2.84

**Table 5:** Model complexity analysis of different methods on 8iVFBv2 datasets.

Methods	Enc/Dec (s/Frame)	FLOPs	#Params.
PCGCv1	4.2/1.8	7.2 G	6.34 M
PCGCv2	1.1/1.0	4.8 G	3.44 M
D-DPCC	1.67/1.67	5.4 G	3.87 M
VPCC	90.7/2.1	-	-
G-PCC (octree)	2.1/0.8	-	-
G-PCC (trisoup)	3.2/1.1	-	-
Proposed	2.57/2.57	6.0 G	4.23 M

## Data Availability

No new data were created or analyzed in this study. Data sharing is not applicable to this article.
